# Functional Characterization of *Populus PsnSHN2* in Coordinated Regulation of Secondary Wall Components in Tobacco

**DOI:** 10.1038/s41598-017-00093-z

**Published:** 2017-03-03

**Authors:** Yingying Liu, Minjing Wei, Cong Hou, Tingting Lu, Lulu Liu, Hairong Wei, Yuxiang Cheng, Zhigang Wei

**Affiliations:** 1 State Key Laboratory of Tree Genetics and Breeding, Northeast Forestry University, Heilongjiang Harbin, 150040 P.R. China; 2 School of Forest Resource and Environmental Science, Michigan Technological University, Houghton, MI 49931 USA

## Abstract

Wood formation is a biological process during which the most abundant lignocellulosic biomass on earth is produced. Although a number of transcription factors have been linked to the regulation of wood formation process, none of them has been demonstrated to be a higher hierarchical regulator that coordinately regulates secondary wall biosynthesis genes. Here, we identified a *Populus* gene, *PsnSHN2*, a counterpart of the *Arabidopsis* AP2/ERF type transcription factor, *SHINE2*. *PsnSHN2* is predominantly expressed in xylem tissues and acted evidently as a high hierarchical transcriptional activator. Overexpression of *PsnSHN2* in tobacco significantly altered the expression of both transcription factors and biosynthesis genes involved in secondary wall formation, leading to the thickened secondary walls and the changed cell wall composition. The most significant changes occurred in the contents of cellulose and hemicellulose that increased 37% and 28%, respectively, whereas the content of lignin that decreased 34%. Furthermore, PsnSHN2 activated or repressed the promoter activities of transcription factors involved in secondary wall biosynthesis and bound to five *cis*-acting elements enriched in the promoter regions of these transcription factors. Taken together, our results suggest *PsnSHN2* coordinately regulate secondary wall formation through selective up/down-regulation of its downstream transcription factors that control secondary wall formation.

## Introduction

Secondary walls in the form of wood and fibers are most abundant biomass produced by vascular plants, and are the most important biomaterials that meet a variety of needs of humans for fibers, textiles, pulping and paper manufacture, and bioenergy^1, 2^. Thus, there has been a tremendous interest in elucidating the underlying molecular mechanisms governing secondary wall formation^3–8^. The secondary wall formation is a developmental process that involves highly coordinated expression of secondary wall biosynthesis genes, which are known to be regulated by a cascade of transcription factors (TFs). Therefore, it is a central task to understand how TFs regulate the secondary wall formation^2, 7–13^. It has been demonstrated that a few NAC domain TFs function as high hierarchical regulators controlling the secondary wall biosynthesis program through activating a nexus of intermediate-level TFs mostly are MYBs^9, 10, 13–15^. These intermediate TFs in turn activate some low-level MYBs that directly regulate secondary wall biosynthesis genes^12, 16^. At the same time, molecular and genomic studies in tree species have also revealed the existence of a hierarchical transcriptional regulation network that consists of a set of TFs, which regulate wood formation in the similar hierarchical fashion as their counterparts in *Arabidopsis thaliana* do^2, 4, 5, 16–19^. Among them, the best-characterized TFs are, for example, *Populus trichocarpa* wood-associated NAC domain TFs (*PtrWNDs*), *Eucalyptus grandis WND1* (*EgWND1*) and *Populus deltoids MYB21* (*PdMYB21*)^5, 6, 16, 17^. Such a multilayered regulatory network implicates the existence of some high hierarchical regulators that can be tuned to alter different secondary wall biosynthesis pathways in order to optimize secondary wall composition^2, 3, 7, 9, 11, 12, 20^. This is a big leap in understanding the molecular mechanisms of wood formation. For this reason, it is important to identify new high hierarchical TFs to authenticate this model^7^.

In plants, APETALA2/ethylene-responsive element-binding proteins (AP2/EREBP) are one of the largest TF families^21^. This super family is characterized by the conserved AP2/ERF DNA-binding domain of 60–70 amino acid residues^22^, and plays a key role in regulating developmental processes, hormonal signal transductions, and environmental responses^23^. Recently, the AP2/EREBP domain transcription factors are also found to participate the secondary wall formation in the developmental stem of *Medicago truncatula* ^24^. There are three *A. thaliana* SHINE genes, *AtSHN1*, *AtSHNE2*, and *AtSHN3*, which belong to ERF-B6 clade of AP2/ERF family and harbor more other two exclusive motifs: “mm” (middle domain, with approximate 61 amino acids) and “cm” (c-terminal domain, containing approximate 10 amino acids)^25–27^. *AtSHN2* is highly precise and temporarily coordinated in anther and silique dehiscence zones^28^. Moreover, *AtSHN2* was previously shown to be involved in wax/cutin lipid regulation and drought tolerance in *Arabidopsis* ^25, 26^. Following these findings, Ambavaram *et al*. demonstrated that *AtSHN2* directly regulates NAC and MYB TFs, and modification of *AtSHN2* coordinately alters cell wall components deposition, causing a 34% increase in cellulose and a 45% decrease in lignin content without any obvious detriment on plant development in the overexpression transgenic lines of rice (*Oryza sativa*)^29^. In *E. grandis*, two SHINE TFs (*EgrSHN1* and *EgrSHN2*) contain all conserved motifs and characteristic features of SHINE family, but their functions in the wood formation have not been characterized^30^. In fact, it is still unknown whether SHINE family genes in trees have functions in the secondary wall formation.

In this study, we investigated the roles of *SHN2* of *Populus simonii* × *Populus nigra* (*PsnSHN2*) in secondary wall formation, and its biological functions in regulating cellulose, hemicellulose and lignin biosynthesis. We showed that ectopic expression of *PsnSHN2* in tobacco led to thickened secondary walls through a coordinated regulation of cellulose, hemicellulose and lignin biosynthesis. We observed the altered expression of some key secondary wall-associated TFs and secondary wall biosynthesis genes in *PsnSHN2* tobacco transgenic lines. Our results indicate that *PsnSHN2* is a high hierarchical TF that governs the secondary wall formation, and is more importantly capable of coordinately regulating cellulose, hemicellulose, and lignin biosynthesis pathways.

## Results

### Isolation and characterization of the *PsnSHN2* from *P. simonii* × *P. nigra*

**Figure 1:**
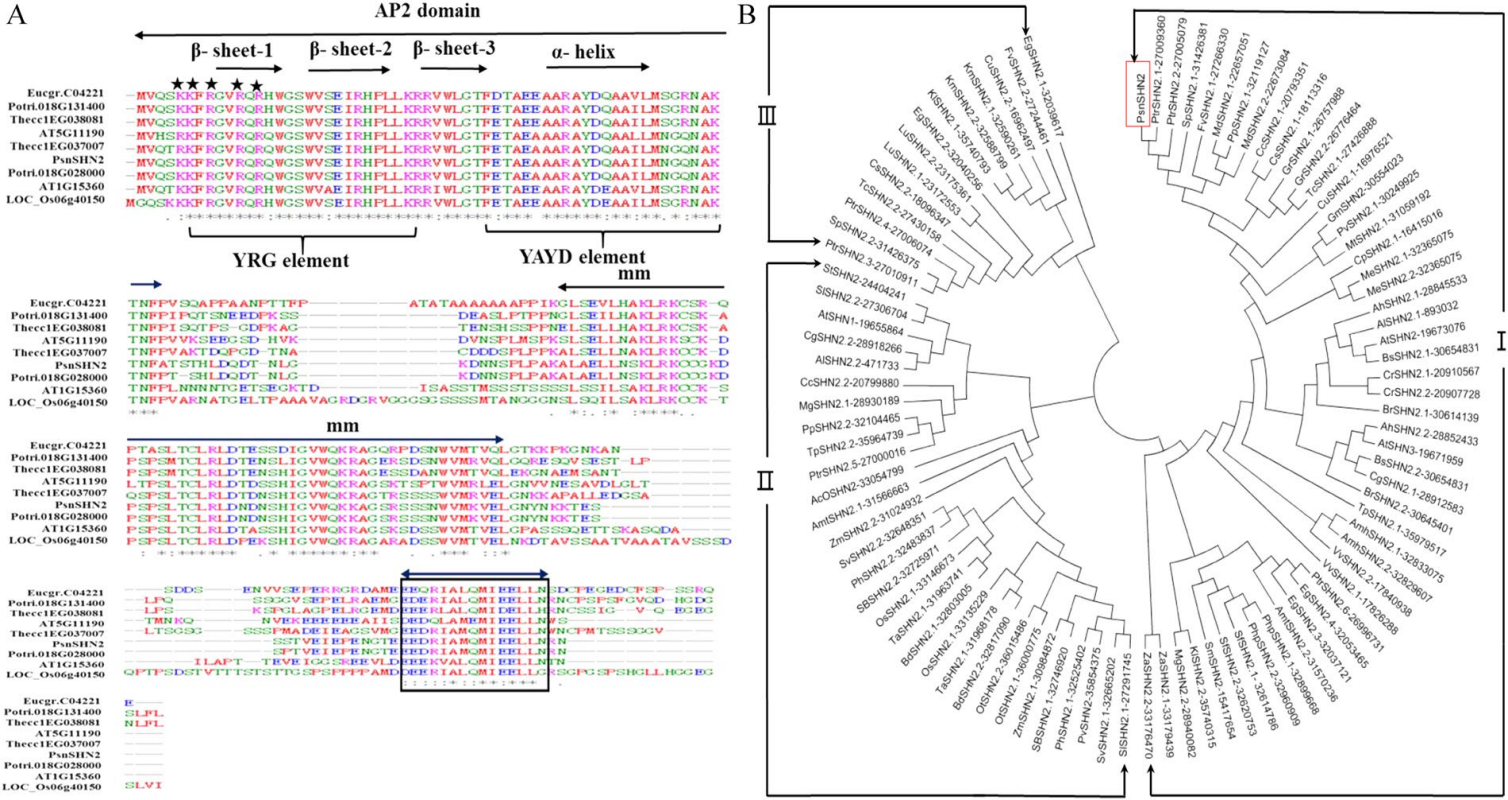
Protein sequence alignment and phylogenetic analysis of *PsnSHN2*. (**A**) PsnSHN2 was aligned with SHINE proteins from *Populus trichocarpa* (Potri.018G028000 and Potri.018G131400), *Eucalyptus globulus* (Eucgr.C04221), *Theobroma cacao* (Thecc1EG038081 and Thecc1EG0370070), *Oryza sativa* (LOC_Os06g40150) and *Arabidopsis thaliana* (AT1G15360 and AT5G11190). The conserved YRG and RAYD elements in AP2 domain are indicated by brackets. α helix is indicated by an arrow; cm stands for c-terminal domain while mm represents middle domain; Asterisks mark the putative domains for nuclear localization signals; the boxed region indicates the putative activation domain. (**B**) Phylogenetic analysis of PsnSHN2 with other SHINE proteins. 93 SHINE genes of 44 species are available in Supplemental file. PsnSHN2 is shown in a red rectangle. The number after short dash is gene ID number from the phytozome database.

We isolated the full length cDNA sequence encoding a protein of 179 amino acid residues, which is 100%, 90.3%, and 95.2% identical to the *P. trichocarpa* putative SHINE protein (27009360), *E. grandis* SHN2 (32053465) and *A. thaliana* SHN2 (19673076), respectively. Its protein sequence contains all the SHINE protein domains that include an AP2 DNA binding domain, a “mm” domain and a “cm” domain (Fig. 1A). The AP2 domain of PsnSHN2 has all the characteristic elements, namely the YRG and RYAD, and the structure of three β-sheets and the α-helix (Fig. 1A). Thus, it was given the nomenclature of *PsnSHN2*, in resemblance to *AtSHN2*. In addition, PsnSHN2 contains a basic N-terminal region that might serve as a nuclear location signal (NLS), and an acidic C-terminal region that might act as an activation domain (Fig. 1A).

Phylogenetic analysis of *PsnSHN2* together with 93 *SHINE* genes with full-length protein sequences from 44 species revealed there are three large phylogenetic classes of SHINE proteins (Fig. 1B). PsnSHN2 was located in the Clade I, the largest one with 50 SHINE proteins from 29 species that were distributed in 12 sub-clades of different levels and branches. The Clade II and III comprised 30 and 14 proteins from 21 and 10 species, respectively, which include a sub-clade where woody trees and shrub species are present. It was noteworthy that the proteins from the same species were not necessarily located in the same sub-clades, and the proteins from both monocotyledonous and dicotyledonous species could present in the same sub-clades. Such results indicated that the evolution of SHINE proteins is not aligned well with the evolution of cotyledons. In addition, phylogenic result showed that the PsnSHN2 was in the same clade with AtSHN2 (Fig. 1B), indicating it may play a role in secondary wall formation, as what AtSHN2 does^29^.

### Expression pattern, subcellular localization and transcriptional activation activity of *PsnSHN2*

**Figure 2:**
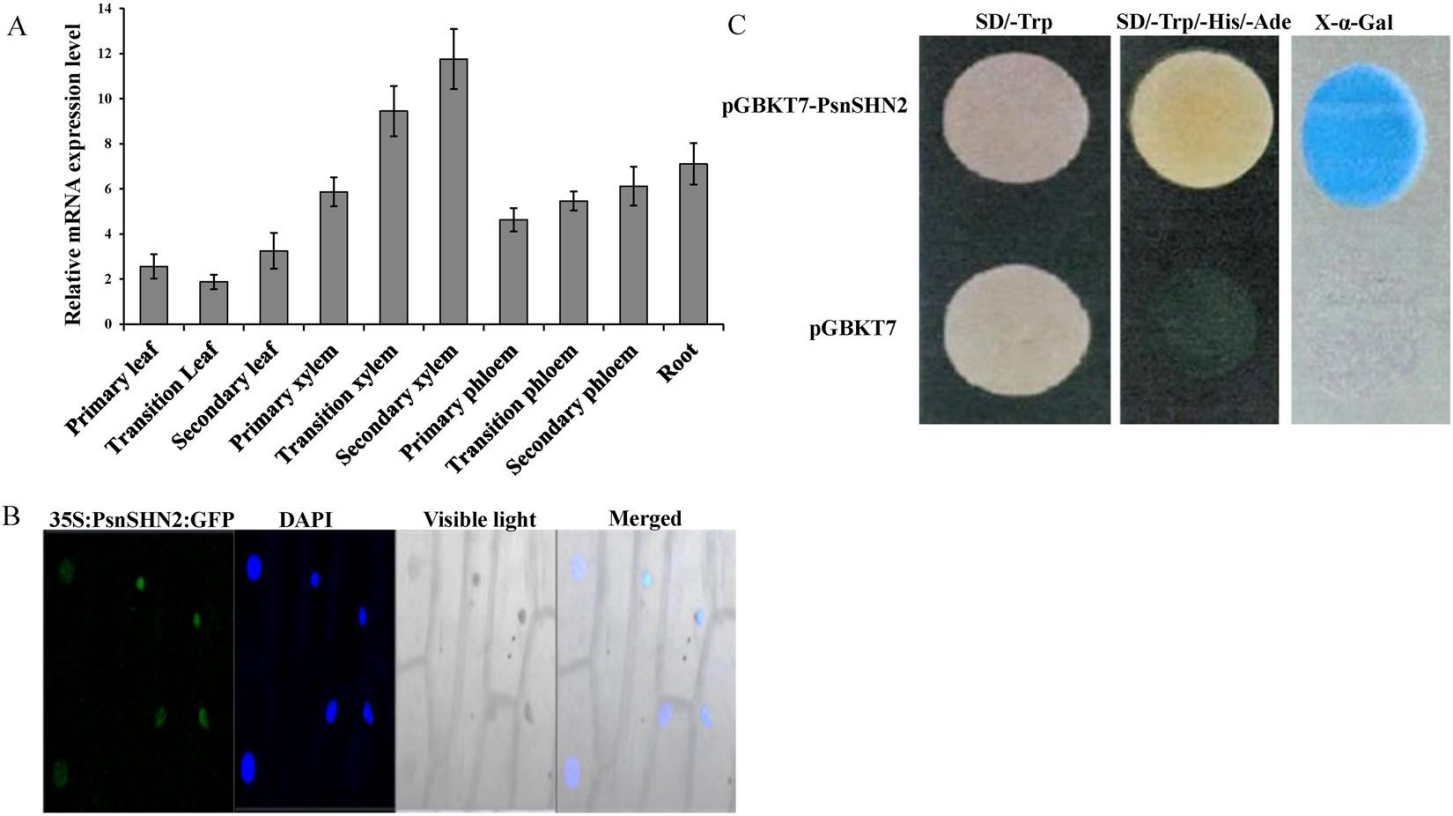
Expression patterns, subcellular localization and transcriptional activity of *PsnSHN2*. (**A**) qRT-PCR analysis of the expression levels of *PsnSHN2* in different poplar tissues. *Actin* was used as a control. Error bars represent the standard deviation (SD) of three biological replicates. (**B**) Subcellular localization of PsnSHN2. Confocal images show the localization of PsnSHN2-GFP in the nuclei of onion epidermal cells. DAPI, a nuclear staining dye; Merged: The merged images of bright-field, GFP and DAPI staining. (**C**) Transcriptional activation analysis of PsnSHN2 fused with the GAL4 DNA binding domain (GAL4DB) in yeast shows its potential to activate the expression of the His-3 and X-α-Gal reporter genes.

To investigate whether *PsnSHN2* is associated with secondary wall biosynthesis, we analyzed its expression levels in different tissues of poplar by qRT-PCR. The result showed *PsnSHN2* was differentially expressed at detectable levels in all tissues as examined (Fig. 2A). The relative levels of the *PsnSHN2* transcripts in the secondary and transition xylem were the higher than any other studied tissues, whereas the expression in the transition leaves is the lowest. The preferential expression in developing secondary xylem tissues suggests that *PsnSHN2* is involved in secondary wall biosynthesis.

The presence of a nuclear localization signal at the N-terminal region of the PsnSHN2 may indicate that the protein is likely to localize in the nuclei (Fig. 1A). To verify this, the *PsnSHN2* coding region was fused to the N-terminus of the GFP gene under the control of the CaMV 35S promoter and transferred into onion epidermal cells using the particle gun bombardment. Localization of the fusion protein was then visualized with a fluorescence confocal microscope. As seen in Fig. 2B, the PsnSHN2-GFP fusion protein was exclusively colocalized to DAPI-stained nuclei, indicating the *PsnSHN2* encodes a nuclear-localized protein.

To examine whether PsnSHN2 can activate transcription, we fused *PsnSHN2* with the GAL4 DNA-binding domain and test its potential to activate the reporter gene expression in yeast. It was found that PsnSHN2 could activate the expression His3 and X-α-Gal reporter genes (Fig. 2C), indicating it is indeed a transcriptional activator.

### The potential of PsnSHN2 as a higher hierarchical TF

**Figure 3:**
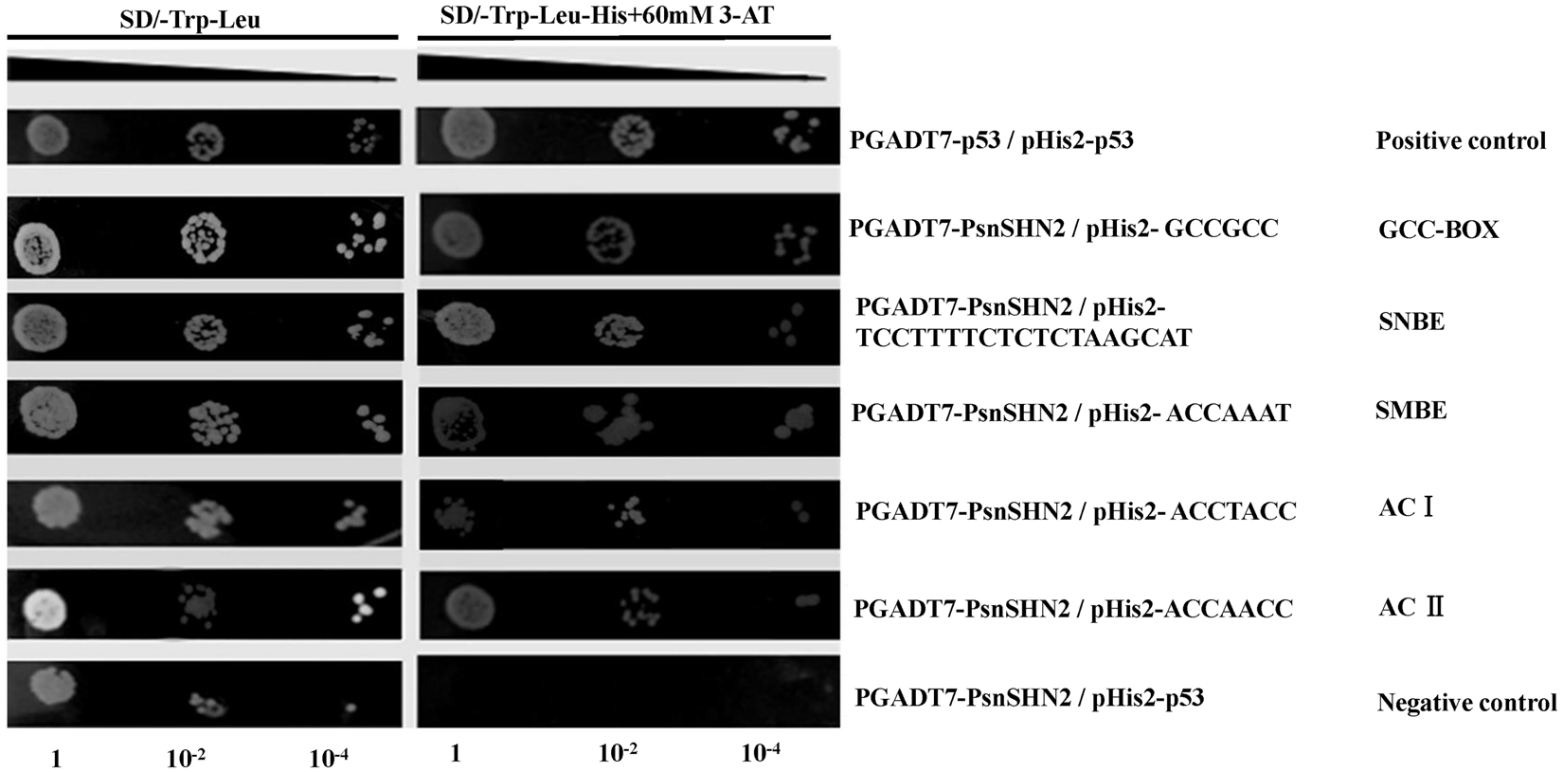
Yeast one-hybrid assay of PsnSHN2′s potential to bind to *cis*-acting elements. The pHIS2-p53/pGADT7-p53 and pHIS2-p53/pGADT7-PsnSHN2 were used as the positive and negative control, respectively. 1, 10^−2^ and 10^−4^ represent solution dilution ratio of transformed Y187 yeast cells.

In order to test whether PsnSHN2 can function as a higher hierarchical TF in the secondary wall biosynthesis process as AtSHN2 does in rice, a yeast one-hybrid assay was performed to test the potential of PsnSHN2 binding to *cis*-acting elements including GCC box, SNBE, SMBE, ACI and ACII element that are often present in the upper streams of TFs (Table S9) present in the hierarchical transcriptional network governing secondary wall formation. The result manifested that PsnSHN2 had obvious binding affinities to these five *cis*-acting elements (Fig. 3).

### Changes in the thickness and composition of the secondary walls caused by *PsnSHN2* overexpression in tobacco

**Figure 4:**
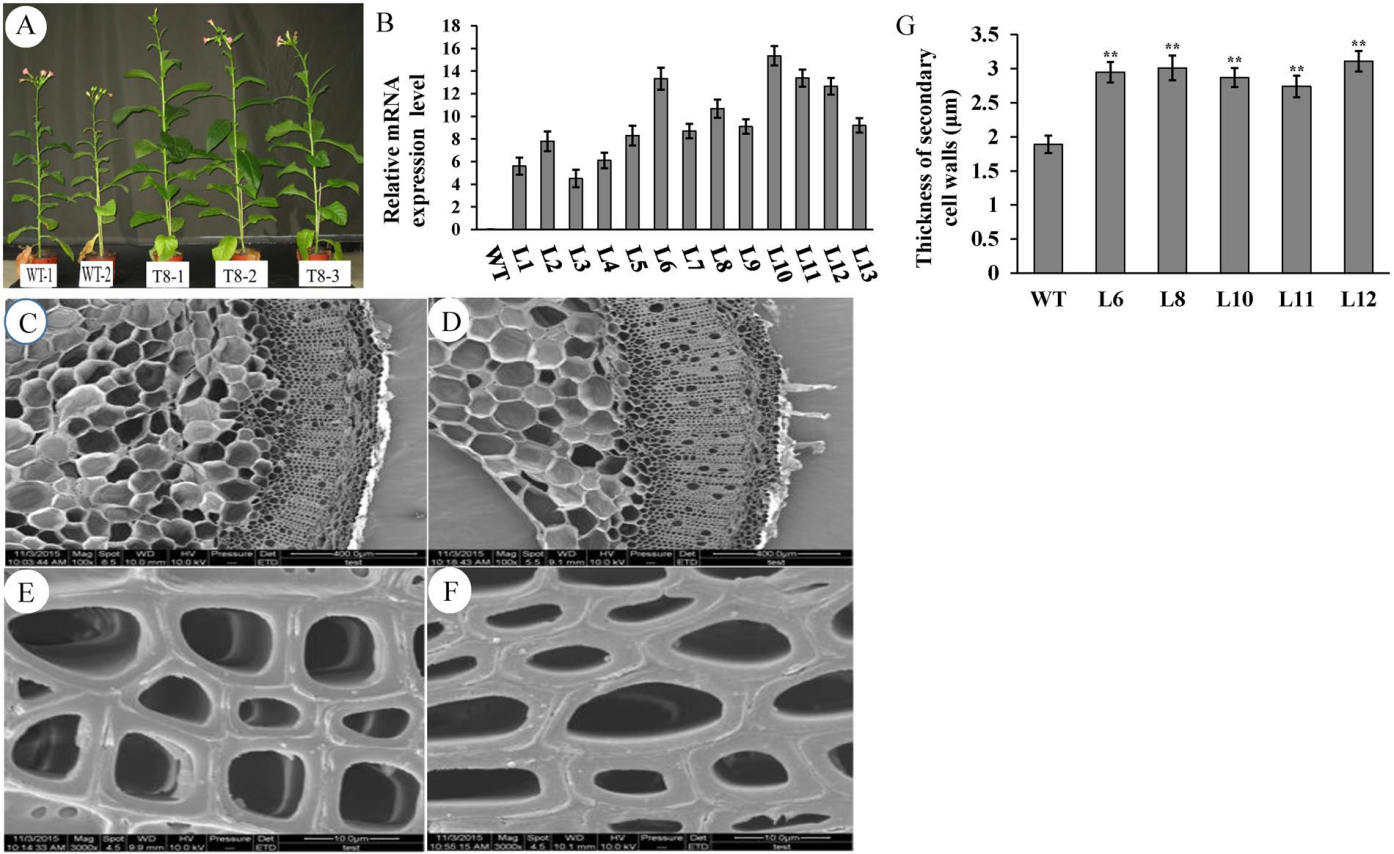
Effect of *PsnSHN2* overexpression on the secondary wall thickness in the stems of *PsnSHN2* transgenic lines. (**A**) Three-month-old wild-type (left) and *PsnSHN2* transgenic lines (middle and right). (**B**) The expression levels of *PsnSHN2* in transgenic lines. Error bars represent SD of three biological replicates. (**C–F**) The scanning electron microscope of cross stem sections of wild-type (**C,E**) and *PsnSHN2* transgenic lines (**D,F**). (**G**) The secondary wall thickness of cross stem sections of *PsnSHN2* transgenic lines. Error bars represent SD of three biological replicates. Asterisks indicate levels of significance (*t* test; *P < 0.05, **P < 0.01).

**Figure 5:**
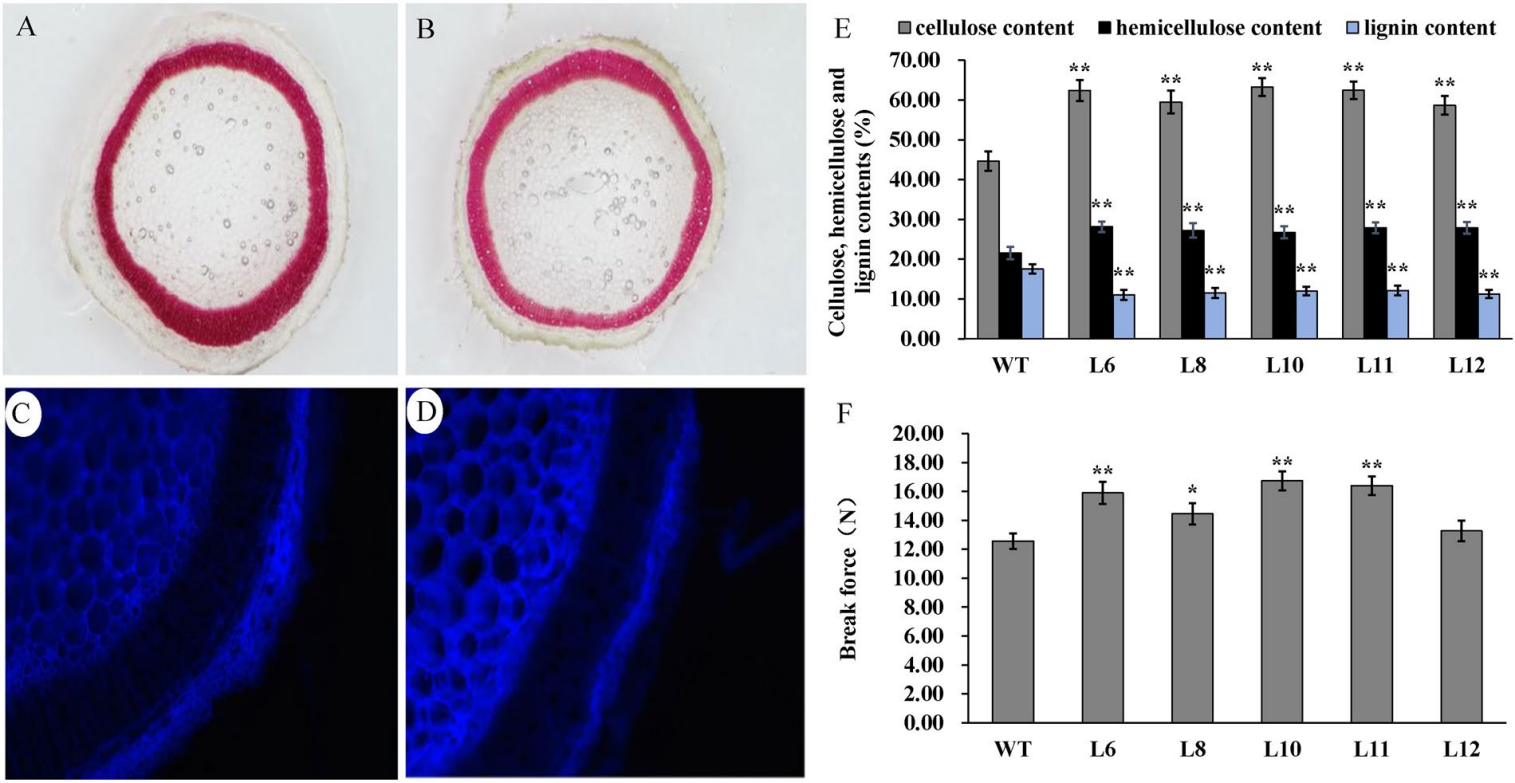
Effects of *PsnSHN2* overexpression on the secondary wall deposition and breaking force in the stems of *PsnSHN2* transgenic lines. (**A,B**) Phloroglucinol-HCl staining (red color) stem sections of *PsnSHN2* transgenic lines (**A**) and wild-type (**B**). (**C,D**) Calcofluor white staining (blue color) stem sections of *PsnSHN2* transgenic lines (**C**) and wild-type (**D**). (**E**,**F**) The contents of cellulose, hemicellulose and lignin (**E**) and the breaking force in stems of *PsnSHN2* transgenic lines (**F**). Error bars represent SD of three biological replicates. Asterisks indicate levels of significance (*t* test; *P < 0.05, **P < 0.01).

We obtained T2 *PsnSHN2* tobacco transgenic lines through hybridization of T1 generations. All *PsnSHN2* lines exhibited more vigorous growth than wild-type (Fig. 4A). We chose those *PsnSHN2* transgenic lines, L6, L8, L10, L11 and L12, which had higher expression levels of *PsnSHN2* (Fig. 4B). To investigate the contribution of *PsnSHN2* overexpression to the secondary wall biosynthesis, we measured the thicknesses and three major components of secondary wall in the stems of *PsnSHN2* transgenic lines. The scanning electron microscope of stem cross-sections and thickness of secondary wall analysis revealed that the secondary wall thicknesses increased 55% in the *PsnSHN2* transgenic lines than those in wild-type (Fig. 4C–G). To identify which components (e.g., cellulose, lignin, or hemicellulose) gave rise to the increased thicknesses of the secondary walls, the stem cross-sections were histochemically stained with phloroglucinol-HCl and calcofluor white solutions. Phloroglucinol stain reacts with coniferaldehyde groups in lignin, and the color intensity reflects the total lignin content, while calcofluor white reacts with cellulose and make it display fluorescence staining. The results clearly showed a decrease in lignin content and an increase in cellulose content in the stems of *PsnSHN2* transgenic lines in comparison with wild-type (Fig. 5A–D). To further assess the alternation of secondary wall composition, the chemical analysis of the stem composition was performed using an automatic fiber analyzer and the results showed a 37% increase in cellulose, a 28% increase in hemicellulose, and a 34% reduction in lignin (Fig. 5E). Owing to these changes, the break force of *PsnSHN2* transgenic lines measured was on average 22% higher than that of the wild-type (Fig. 5F). Taken together, these results support the hypothesis that *PsnSHN2* coordinately regulates the biosynthesis pathways of cellulose, hemicellulose, and lignin.

### Lignocellulosic pathway genes and TFs induced by *PsnSHN2* overexpression in tobacco

**Figure 6:**
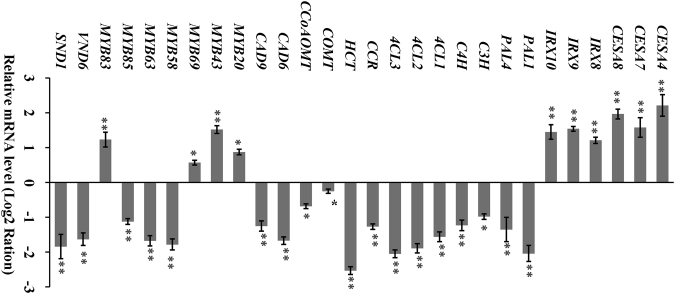
Expression analysis the TF genes and secondary wall biosynthesis genes in *PsnSHN2* transgenic lines. Secondary wall biosynthesis genes for cellulose (*CESA4*, *CESA7*, and *CESA8*), hemicellulose (*IRX8*, *IRX9*, and *IRX10*), and lignin (*PAL1*, *PAL4*, *C3H*, *C4H*, *4CL1*, *4CL2*, *4CL3*, *CCR*, *HCT*, *COMT*, *CCoAOMT*, *CAD6*, and *CAD*9). *MYB20*, *MYB43*, *MYB69*, *MYB58*, *MYB63*, *MYB85*, *MYB83*, *VND6,* and *SND1* are known TFs that control the secondary wall formation. Error bars represent SD of three biological replicates. Asterisks indicate levels of significance (*t* test; *P < 0.05, **P < 0.01).

The finding that overexpression of *PsnSHN2* induced ectopic deposition of secondary wall and coordination alternation the secondary wall composition prompted us to determine the expression levels of secondary wall biosynthesis pathway genes in the stems of *PsnSHN2* transgenic lines. The results from qRT-PCR analysis demonstrated that the transcript levels of tobacco lignin biosynthesis pathway genes (including *PAL1*, *PAL4*, *C3H*, *C4H*, *4CL1*, *4CL2*, *4CL3*, *CCR*, *HCT*, *COMT*, *CCoAOMT*, *CAD6*, and *CAD9*)^31^ were significantly repressed in the stem of *PsnSHN2* transgenic lines (Fig. 6). On the other hand, the expression of a number of cellulose and hemicellulose biosynthesis pathway genes, such as three CEAS genes (*CESA4*, *CESA7*, and *CESA8*)^32^ and three CESA-like genes (*IRX8*, *IRX9*, and *IRX10*)^33^, were significantly up-regulated in the stems of *PsnSHN2* transgenic lines compared with wild-type (Fig. 6).

To unravel the molecular mechanism underlying the altered expression of secondary wall biosynthesis genes in the stems of *PsnSHN2* transgenic lines, we further analyzed the expression levels of a number of TFs known to control these secondary wall biosynthesis genes using qRT-PCR. The results demonstrated that the transcript abundance of NAC TFs (*SND1* and *VND6*), and MYB TFs (*MYB58*, *MYB63*, and *MYB85*), which specifically activate lignin biosynthesis pathway genes^34^ (Fig. 6), were significantly repressed in *PsnSHN2* transgenic lines (Fig. 6). On the contrary, the expression levels of the one nexus intermediate switch *MYB83* ^11^, and several MYB genes (*MYB20*, *MYB43*, and *MYB69*) that specially activate the cellulose and hemicellulose biosynthesis genes^11^, increased significantly in *PsnSHN2* transgenic lines (Fig. 6).

### Activation of Poplar TFs involved in regulating wood formation by PsnSHN2 in tobacco

**Figure 7:**
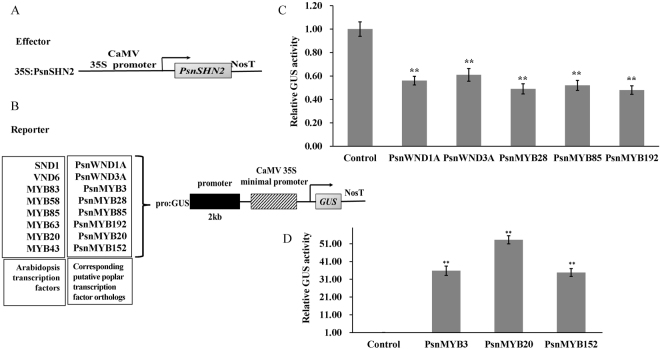
Activation or repression of the promoters of poplar TFs by PsnSHN2. (**A,B**) Diagrams of the effector and reporter constructs. (**C,D**) The expression of the *GUS* was repressed and activated by *PsnSHN2*, respectively. GUS activity in tobacco leaves transfected with the reporter construct alone was used as a control and was set to 1. Error bars represent SD of three biological replicates. Asterisks indicate levels of significance of differential expression (*t* test; *P < 0.01).

Based on the fact that *PsnSHN2* induced the expression of a number of TFs that regulate pathway genes of secondary wall biosynthesis in transgenic tobacco, we investigated whether it can directly control poplar counterparts. We performed transcriptional activation assays to analyze whether the promoters of these poplar TFs could be activated or repressed by *PsnSHN2* in tobacco leaves. The 2-kb proximal promoter regions amplified from *P. simonii* × *P. nigra* genomic DNA were linked to the GUS reporter gene to create the reporter constructs, and the full-length cDNA of *PsnSHN2* was ligated to the CaMV 35S promoter to generate the effector construct (Fig. 7A,B). The reporter and effector constructs were co-transfected into tobacco leaves, together with 35S:LUC that was primarily used for normalizing the transformation efficiency values. The subsequent assay of the GUS activity in the transfected leaves demonstrated that PsnSHN2 significantly repressed the activities of *PsnWND1A*, *PsnWND3A*, *PsnMYB28*, *PsnMYB85*, and *PsnMYB192* promoters (Fig. 7C), and significantly activated the activities of the *PsnMYB3*, *PsnMYB20*, and *PsnMYB152* promoters (Fig. 7D).

## Discussion

The biosynthesis of secondary wall involves a coordinated expression of secondary wall biosynthesis genes regulated by a cascade of TFs^2, 7, 9, 11, 12, 35^. To look into the molecular mechanism of wood formation and develop molecular means for genetic improvement of wood productivity and quality, it is necessary to continuously identify new master regulators or switches whose changes can boost and suppress low hierarchical TFs and further downstream lignocellulosic pathway genes in a differential manner^5, 6, 10, 13, 17^. The AP2/EREBP gene family have been reported to regulate plant secondary growth^24^, development and environmental responses^23^, which indicate that AP2/EREBP family genes may have pleiotropic effects on multiple biological processes, and may potentially serve as high hierarchical TFs. For example, as a member of AP2/EREBP family genes, *AtSHN2* has been reported to play a role not only in coordinated regulation of lignin and cellulose/cell wall biosynthesis in transgenic rice^29^, but also in cuticle (cuticular wax and cutin) formation in response to drought^25, 26^. As a high degree of conservation exists between transcription factors involved in the secondary wall formation between herbaceous and woody plants^36^, it is reasonable to propose that the *SHN2* gene in poplar may function in the similar way in regard to the secondary wall formation. In order to identify a new TF involved in secondary wall formation in poplar like *AtSHN2*, we have cloned and characterized *PsnSHN2* from *P. simonii* × *P. nigra*, an economically important tree species in the northeast region of P.R. China. The PsnSHN2 showed a high amino acid similarity with AtSHN2 in *A. thaliana*, and is clustered into the same clade of with AtSHN2. The previous studies about the necessity of valine (V) at the 14^th^ position and glutamic acid (E) at 19^th^ position in the AP2/ERF domain proteins for binding to GCC box are evidently controversial^22, 37, 38^. One example is that several ERF proteins containing alanine (A) and aspartic acid (D) at the 14^th^ and 19^th^ amino acids can bind to GCC box^39, 40^. In this study, PsnSHN2 containing Glutamine (Q) at the 14^th^ position and V at the 19^th^ position instead of V at the14^th^ and E at the 19^th^ or A at the 14^th^ and D at the 19^th^ can bind to GCC box (Fig. 3).

PsnSHN2 contains an acidic C-terminal region (cm), as shown in rice and durum wheat, which act as a transcriptional activation domain^37, 41^. Our results of yeast two-hybrid analysis revealed that PsnSHN2 has transcriptional activity (Fig. 2C), and yeast one-hybrid analysis indicated that PsnSHN2 could bind to five important *cis*-acting elements that are often present in proximal regions of wood formation genes (Fig. 3). In addition, the presence of NLS indicates that PsnSHN2 might be targeted to the nuclei (Fig. 1), which was further corroborated by localization of recombinant PsnSHN2-GFP protein to the nuclei of onion epidermal cells (Fig. 2B). Moreover, *PsnSHN2* is predominantly expressed in developing xylem tissues of poplar (Fig. 2A). Based on these results, we propose that *PsnSHN2* appears to act as a high hierarchical TF that exerts strong regulation of secondary wall formation, having a resemblance to *AtSHN2* that functions in rice secondary wall formation^29^.

Our finding that the ectopic expression of *PsnSHN2* led to a coordinated regulation between repression of lignin and activation of cellulose and hemicellulose biosynthesis, which eventually resulted in the thickened secondary wall (Fig. 4D,F). The declination of lignin content caused by *PsnSHN2* overexpression is somewhat similar to the down-regulation of *PAL*, *4CL*, *CAD* and their upstream TFs in *Arabidopsis* and other plants^42^. Similarly, plants expressing both antisense *4CL* and sense *CALd5H* have been reported to have 52% less lignin and 30% more cellulose^43^. On the contrary, the rice *bc1* mutant and maize *bk2* mutant, which all encode COBRA-like protein, have been shown to contain more lignin and less cellulose^44, 45^. We noticed that the changes in contents of cellulose, hemicellulose and lignin did not adversely affect growth of the *PsnSHN2* transgenic lines, as evidenced by the fact that *PsnSHN2* transgenic lines displayed normal plant phenotype, maturity, and seed yield in greenhouse condition (Fig. 4A). Surprisingly, the *PsnSHN2* transgenic lines even grew bigger and more vigorously than wild-type, for example, much larger leaves and bigger diameters of stems, and taller height, as well as more biomass (Fig. S1). Such phonotypes with increased cellulose and reduced lignin have been reported in the *4CL* antisense transgenic aspen lines, which also exhibit better growth^46^. Based on these facts, we speculated that the increased growth in all dimensions in the *PsnSHN2* transgenic lines arose from the reduction in lignin because lignification in plants often has strong inverse correlation with growth^47^. In addition, the breaking force of the stems of *PsnSHN2* transgenic lines became stronger than that of wild-type (Fig. 5F). The previous study found the tensile or bending strength of maize was shown to correlate with the cellulose content^48^. Therefore, the *PsnSHN2* transgenic lines with increased the contents of cellulose and hemicellulose probably offset some reduction in mechanical strength caused a decrease in lignin content. It is worth to mention that the thickened secondary wall in *PsnSHN2* transgenic lines also contributed to the stem tensile strength.

In summary, the evidence from three experiments consistently supports *PsnSHN2* as a higher hierarchical TF exerted coordinate regulation of the secondary wall component mainly through regulation low hierarchical TFs in transcriptional network governing secondary wall formation. Evidence includes (1) *PsnSHN2* overexpression in tobacco caused the low-level TFs to be activated or repressed, such as NACs (*SND1* and *VND6*) and MYBs (*MYB58*, *MYB63*, and *MYB85, et al.*). (2) Yeast one-hybrid assay showed PsnSHN2 could bind to *cis*-acting elements, which are often present in the proximal regions of TFs controlling secondary wall biosynthesis genes. (3) PsnSHN2 could directly transactivate or suppress the expression of low hierarchical TFs involved in wood formation. For example, PsnSHN2 could repress the poplar master switches TFs, *PsnWND1A* and *PsnWND3A*. At a relatively high hierarchical level, *PsnSHN2* could regulate and/or coordinate multiple pathways through controlling the low-level TFs in a top-down fashion.

Although our finding that *PsnSHN2* played a high hierarchical role in controlling the secondary wall formation may provide some basis for further our understanding on the regulation of wood formation in woody plants, it is noteworthy to mention that we cannot deduce the functions of *PsnSHN2* in the wood formation in tree species through directly applying the knowledge of *PsnSHN2* gained from the overexpression transgenic tobacco lines even though the conservative mechanisms exist in regulating vascular plant secondary growth between herbaceous and wood plant species^16^. Although the stem structures of tobacco plants resemble to those of tree species^49^, there are some distinct features between xylem tissues in woody and herbaceous tobacco^49^, implicating that some unique regulatory mechanisms are present in tree species in controlling secondary wood formation^50^. Further, it has been reported that some orthologous TFs have marginally or drastically diverged function^51, 52^, and even the same TF can have different effects on secondary growth in different transgenic plants^29, 53^. Therefore, it is still necessary to characterize the functions of the *PsnSHN2* in wood formation of tree species. Nevertheless, the activity of *PsnSHN2* in the herbaceous model tobacco shows that it has a potential role in engineering the cellulose-lignin composition and content in herbaceous plants or other suitable biomass producers. The *PsnSHN2* master regulator can also be used as a tool for unveiling the regulatory pathway in secondary wall formation.

## Materials and Methods

### Plant materials and RNA extraction

Two-year-old hybrid (*P. simonii* × *P. nigra*) trees were propagated and planted in a mixture of turfy peat and sand (2:1 v/v) in a greenhouse owned by Northeast Forestry University, Harbin, Heilongjiang Province, P.R. China. The primary shoot leaves, transition leaves, secondary leaves, primary xylem, transition xylem, secondary xylem, primary phloem, transition phloem, secondary phloem and roots were collected and immediately frozen in liquid nitrogen and stored at −80 °C. The RNA was isolated according to a previously published method^54^ and then treated with DNase I (Qiagen) to remove any residual of genomic DNA.

### Cloning PsnSHN2 gene from* P. simonii *× *P. nigra*

Five micrograms of total RNA was used for the synthesis cDNA using SuperScript II Reverse Transcriptase (Invitrogen) and oligo (dT) 16 primers, according to the manufacturer’s instructions. The full cDNA fragment encoding *PsnSHN2* was amplified from *P. simonii* × *P. nigra* with gene-specific primers (Table S1). The PCR product was cloned into the pMD18-T cloning vector (TaKaRa), and then transformed into *Escherichia coli* cells (DH5α) for massive sequencing.

### Sequence comparisons and phylogenetic analysis

Multiple sequence alignments were carried on using ClustalW2 (available in http://www.ebi.ac.uk/Tools/msa/clustalw2/) with default setting. SHINE homologous gene sequences from *P. trichocarpa* (Potri.018G028000 and Potri.018G131400), *Eucalyptus globulus* (Eucgr.C04221), *Theobroma cacao* (Thecc1EG038081 and Thecc1EG0370070), *O. sativa* (LOC_Os06g40150) and *A. thaliana* (AT1G15360 and AT5G11190) were retrieved in Phytozome Database (http://www.phytozome.net/) using TBLASTN with the *PsnSHN2* sequence being query. A total of 93 putative plant SHN2 protein sequences from 44 species (Supplementary file) were aligned with PsnSHN2 using ClustalW2. A phylogenetic tree was constructed by the MEGA 5.0 using the neighbor-joining method with complete deletion; 1,000 replicates were used for bootstrap analysis, and the cutoff value was set to 50%.

### Subcellular localization

The full-length coding region of *PsnSHN2* without the termination codon was amplified using specific primers (Table S2) and ligated in frame to the N–terminal of GFP driven by CaMV 35S promoter in pGWB5 vector to generate the 35S:SHN2-GFP plant expression vector. The plasmids were delivered into onion epidermal cells by Gene Gun (GJ-1000). The onion skin tissues were stained with DAPI 24 h after bombardment, and then photographed with confocal microscopy (Leica TCS SP5).

### Transcriptional activation assay

The transcriptional activation of PsnSHN2 on putative targets was corroborated using the yeast two–hybrid system. The complete CDS of *PsnSHN2* was amplified using specifically designed primers (Table S3). The amplified fragments were fused in-frame to the GAL4 DNA binding domain in the pGBKT7 vector to generate the pGBKT7-PsnSHN2 construct. The pGBKT7-PsnSHN2 and the pGBKT7 blank vector (as negative control) were transformed into AH109 yeast cells independently. The transformed AH109 yeast cells were plated onto SD/-Trp (growth control), SD/-Trp/-His/-Ade and X-α-Gal media and incubated on at 30 °C for 3–5 days to identify the transcriptional activation.

### Analysis of the downstream *cis*-elements of the *PsnSHN2*

Three tandem copies of the secondary NAC binding element (SNBE)^8, 55^, secondary MYB binding element(SMBE)^8^, ACI and ACII*cis*–acting elements^34^, and GCC box motif^29, 56^ (Table S4) were inserted into pHIS2 (Clontech) upstream of the reporter HIS3, respectively. The full CDS of *PsnSHN2* amplified used primers in Table S4 was inserted into pGADT7-Rec2 as the effector vector. Both constructs were cotransformed into Y187 yeast cells, which were plated onto SD medium minus Trp, Leu, and His plus 60 mM 3-amonotrizole to test the expression of the His3 gene at 30 °C for 3–5 days. The cotransformation of pHIS2-p53 and pGADT7-p53 were used as a positive control, and the pHIS2-p53 and pGADT7-PsnSHN2 were used as a negative control. The interactions of these sequences with the PsnSHN2 were studied using yeast one-hybrid analysis as aforementioned.

### Transformation tobacco plant

The full length CDS of *PsnSHN2* was amplified with the primers listed in Table S5 from pMD18-T cloning vector. Then, the amplified fragments and pROKII vector were double digesting with BamHI and KpnI at same time. The gel-purified *PsnSHN2* and pROKII vector were ligated by T4 ligase. The obtained construct, pROKII-*PsnSHN2,* was transferred into *Agrobacterium tumefaciens* EHA105 using the freeze-thaw method. Transgenic plants were selected on MS medium containing 250 ug/ml kanamycin and 500 ug/ml carbenicillin. The shoots of each transgenic line were cut and cultured until sufficient replicates were generated. All plants were transferred to greenhouse. The T1 seeds were collected from self-pollinated plants and were germinated on MS medium with kanamycin (25 mg/L) to produce T1 generation transgenic plants. These procedures were repeated to obtain the T2 generation seeds. The genomic DNA was extracted from the leaves of one-month-old *PsnSHN2* T2 seedlings using Depure Plant DNA kit (Deaou Biology Company). The genomic DNA was amplified by regular PCR using the pROKII sequencing primers listed in Table S5 to verify whether *PsnSHN2* was integrated into tobacco genome. All tested *PsnSHN2* transgenic lines and wild-type were grown in the greenhouse and used for analyses.

The plastichron index (PI) method was used to determine the stem growth. The first leaf with a length greater than 5 cm was defined as the first leaf and named PI0. Then the leaf immediately below PI0 was defined as PI1. Stem segments between PI5 and PI8 were used for analyzing the secondary wall thickness, histology and anatomy, cell wall chemical composition. Stem between PI3 and PI5 was retained for analyses of RNA transcript abundance.

### Quantitative RT-PCR (qRT-PCR)

Five micrograms of total RNA from different tissues of *P. simonii* × *P. nigra*, as well as stem segments between PI3 and PI5 of *PsnSHN2* lines and wild-type were used for the synthesizing cDNA using SuperScript II Reverse Transcriptase (Invitrogen) and oligo(dT) 16 primers according to the manufacturer’s instructions. Samples were run in triplicate with the SYBR premix ExTaq kit (TaKaRa) and an Applied Biosystems 7500 Real-Time PCR System to determine the critical threshold (Ct).

The *PsnSHN2* expression level in *P. simonii* × *P. nigra* different tissues and in the stem of *PsnSHN2* transgenic lines were detected by qRT-PCR using primers listed Table S6. The changes in the expression of postulated downstream target TFs (such as NACs and MYBs), and pathway genes involved the secondary wall biosynthesis, which are known in previous studies^2, 9, 11, 13, 14, 20, 32, 34, 42^, were also characterized using qRT-PCR using primers listed in Table S7. The downstream target TFs were also inferred from the previous study of *AtSH2* in rice via homology mapping^29^. The relative expression of each gene relative to the reference gene was calculated using the delta-delta CT method^57^. The reference gene was *PsnACTIN1* in poplar and the *NtACTIN 2* in tobacco (*Nicotiana tabacu*m) (Table S6)*.* Each measurement was carried out with three biological replicates, and three technical replicates. The error bars represent SE of the mean fold changes for the three biological replicates.

### Determination of break force

The breaking force needed to break a stem segment into two segments between PI5 and PI8 was calculated using YYD-1 plant stalk analyzer (Zhejiang Top Instrument Co., Ltd.).

### Scanning Electron Microscopy

Stem segments between PI5 and PI8 of *PsnSHN2* transgenic lines and wild-type were used for scanning electron microscopy (S-4800, HITACHI). The secondary wall thicknesses of fibers in the scanning electron microscopy micrographs were quantified in an area of 45 cells using Image J software (http://rsbweb.nih.gov/ij/).

### Histological Analysis

The stem segments between PI5 and PI8 were cut into 0.5-mm-thick sections that were stained for lignin with phloroglucinol-HCl and examined by light microscopy. The cellulose was stained with calcofluor white and examined by fluorescence microscope.

### Determination of the cellulose, hemicellulose, and lignin contents

The stem segments between PI5 and PI8 were ground to a powder after drying. The cellulose, hemicellulose and lignin contents were analyzed according to the Klason procedure^58^ using an ANKOM 2000i Automatic fiber analyzer (Ankom).

### Transactivation Assay

To test if PsnSHN2 can activate the poplar downstream target TFs promoters such as *PsnWND1A*, *PsnWND3A*, *PsnMYB28*, *PsnMYB85*, *PsnMYB192*, *PsnMYB3*, *PsnMYB20* and *PsnMYB152* ^11, 16, 20^, which were chosen based on the expression analysis results of *PsnSHN2* transgenic lines and the previous study of *AtSHN2* in rice^29^, the full coding region of *PsnSHN2* was cloned into pROKII under the control of the CaMV 35S promoter as the effector construct. The reporter construct contained the GUS reporter gene driven by a 2-kb promoter of TFs, which was amplified using the primer listed in Table S8 from the *P. simonii* × *P. nigra* genomic DNA. Both effector and reporter constructs were cotransfected into tobacco leaves by *A. tumefaciens*-mediated transient transformation^59^. Another construct containing the firefly luciferase (LUC) gene driven by the CaMV 35S promoter was also cotransfected for monitoring the transfection efficiency.

After agroinfiltration, plants were covered with a transparent plastic cover and transferred into a growth chamber at 25 °C with 16/8 h light/dark cycle for 2–3 days. The transfection leaves were soaked with 100 mM MG-132 (Wako Pure Chemical) for 6 h. Then, the total protein of leaves was extracted in the extraction buffer^60^. For LUC activity analysis, the LUC substrate (Promega, Madison, WI) was prepared according to the manufacturer’s instructions. 10 ml of sample extract was mixed with 50 ml of substrate, and the LUC activity was measured on a Zylux FB15 luminometer (Fisher Scientific, Pittsburgh, PA). GUS activities were determined by fluorometry with 4-methylumbelliferyl glucuronide as substrate according to Desikan *et al*.^61^. The GUS activity was normalized against the luciferase activity in each transfection, and the data are averages of three biological replicates.

### Statistical Analysis

The student’s *t* test (http://www.graphpad.com/quickcalcs/ttest1.cfm) was used for statistical analysis of the data generated in the experiment. In all analyses, it was found that the quantitative differences between two groups of data in comparison were statistically significant (*P-value < 0.05; **P-value < 0.01).

## Electronic supplementary material

Supplementary file

## References

[1] Plomion C, Leprovost G, Stokes A (2001). Wood formation in trees. Plant Physiol.

[2] Grant EH, Fujino T, Beers EP, Brunner AM (2010). Characterization of NAC domain transcription factors implicated in control of vascular cell differentiation in Arabidopsis and Populus. Planta.

[3] Handakumbura PP, Hazen SP (2012). Transcriptional Regulation of Grass Secondary Cell Wall Biosynthesis: Playing Catch-Up with Arabidopsis thaliana. Front Plant Sci.

[4] Lee C, Teng Q, Zhong R, Ye ZH (2011). Molecular dissection of xylan biosynthesis during wood formation in poplar. Mol Plant.

[5] Ohtani M (2011). A NAC domain protein family contributing to the regulation of wood formation in poplar. Plant J.

[6] Lin YC (2013). SND1 transcription factor-directed quantitative functional hierarchical genetic regulatory network in wood formation in Populus trichocarpa. Plant Cell.

[7] Du J, Groover A (2010). Transcriptional regulation of secondary growth and wood formation. J Integr Plant Biol.

[8] Zhong R, Lee C, Ye ZH (2010). Global analysis of direct targets of secondary wall NAC master switches in Arabidopsis. Mol Plant.

[9] Zhong R, Lee C, Zhou J, McCarthy RL, Ye ZH (2008). A battery of transcription factors involved in the regulation of secondary cell wall biosynthesis in Arabidopsis. The Plant cell.

[10] Mitsuda N (2007). NAC transcription factors, NST1 and NST3, are key regulators of the formation of secondary walls in woody tissues of Arabidopsis. Plant Cell.

[11] Nakano Y, Yamaguchi M, Endo H, Rejab NA, Ohtani M (2015). NAC-MYB-based transcriptional regulation of secondary cell wall biosynthesis in land plants. Front Plant Sci.

[12] Zhong R, Ye ZH (2007). Regulation of cell wall biosynthesis. Curr Opin Plant Biol.

[13] Zhong R, Demura T, Ye ZH (2006). SND1, a NAC domain transcription factor, is a key regulator of secondary wall synthesis in fibers of Arabidopsis. Plant Cell.

[14] Mitsuda N, Seki M, Shinozaki K, Ohme-Takagi M (2005). The NAC transcription factors NST1 and NST2 of Arabidopsis regulate secondary wall thickenings and are required for anther dehiscence. Plant Cell.

[15] Zhong R, Richardson EA, Ye ZH (2007). Two NAC domain transcription factors, SND1 and NST1, function redundantly in regulation of secondary wall synthesis in fibers of Arabidopsis. Planta.

[16] Zhong R, Lee C, Ye ZH (2010). Functional characterization of poplar wood-associated NAC domain transcription factors. Plant Physiol.

[17] Tang X (2015). Poplar PdMYB221 is involved in the direct and indirect regulation of secondary wall biosynthesis during wood formation. Sci Rep.

[18] Tian Q (2013). Functional characterization of the poplar R2R3-MYB transcription factor PtoMYB216 involved in the regulation of lignin biosynthesis during wood formation. PLoS One.

[19] Zhong R, McCarthy RL, Haghighat M, Ye ZH (2013). The poplar MYB master switches bind to the SMRE site and activate the secondary wall biosynthetic program during wood formation. PLoS One.

[20] Zhong R, McCarthy RL, Lee C, Ye ZH (2011). Dissection of the transcriptional program regulating secondary wall biosynthesis during wood formation in poplar. Plant Physiol.

[21] Mizoi J, Shinozaki K, Yamaguchi-Shinozaki K (2012). AP2/ERF family transcription factors in plant abiotic stress responses. Biochim Biophys Acta.

[22] Sakuma Y (2002). DNA-binding specificity of the ERF/AP2 domain of Arabidopsis DREBs, transcription factors involved in dehydration- and cold-inducible gene expression. Biochem Biophys Res Commun.

[23] Feng JX (2005). An annotation update via cDNA sequence analysis and comprehensive profiling of developmental, hormonal or environmental responsiveness of the Arabidopsis AP2/EREBP transcription factor gene family. Plant Mol Biol.

[24] Wang H (2016). Transcriptome analysis of secondary cell wall development in Medicago truncatula. BMC Genomics.

[25] Aharoni A (2004). The SHINE clade of AP2 domain transcription factors activates wax biosynthesis, alters cuticle properties, and confers drought tolerance when overexpressed in Arabidopsis. Plant Cell.

[26] Broun P, Poindexter P, Osborne E, Jiang CZ, Riechmann JL (2004). WIN1, a transcriptional activator of epidermal wax accumulation in Arabidopsis. Proc Natl Acad Sci USA.

[27] Taketa S (2008). Barley grain with adhering hulls is controlled by an ERF family transcription factor gene regulating a lipid biosynthesis pathway. Proc Natl Acad Sci USA.

[28] Shi JX (2011). SHINE transcription factors act redundantly to pattern the archetypal surface of Arabidopsis flower organs. PLoS Genet.

[29] Ambavaram MM, Krishnan A, Trijatmiko KR, Pereira A (2011). Coordinated activation of cellulose and repression of lignin biosynthesis pathways in rice. Plant Physiol.

[30] Marques WL (2013). Identification of four Eucalyptus genes potentially involved in cell wall biosynthesis and evolutionarily related to SHINE transcription factors. Plant Growth Regul.

[31] Raes J, Rohde A, Christensen JH, Van de Peer Y, Boerjan W (2003). Genome-wide characterization of the lignification toolbox in Arabidopsis. Plant Physiology.

[32] Taylor NG, Gardiner JC, Whiteman R, Turner SR (2004). Cellulose synthesis in the Arabidopsis secondary cell wall. Cellulose.

[33] Pena MJ (2007). Arabidopsis irregular xylem8 and irregular xylem9: Implications for the complexity of glucuronoxylan biosynthesis. Plant Cell.

[34] Zhou J, Lee C, Zhong R, Ye ZH (2009). MYB58 and MYB63 are transcriptional activators of the lignin biosynthetic pathway during secondary cell wall formation in Arabidopsis. Plant Cell.

[35] Zhong R, Lee C, Ye ZH (2010). Evolutionary conservation of the transcriptional network regulating secondary cell wall biosynthesis. Trends Plant Sci.

[36] Hussey, S. G., Mizrachi, E., Creux, N. M. & Myburg, A. A. Navigating the transcriptional roadmap regulating plant secondary cell wall deposition. *Frontiers in Plant Science* **4**, doi: ARTN 32510.3389/fpls.2013.00325 (2013). 10.3389/fpls.2013.00325 PMC3756741 24009617

[37] Djemal R, Khoudi H (2015). Isolation and molecular characterization of a novel WIN1/SHN1 ethylene-responsive transcription factor TdSHN1 from durum wheat (Triticum turgidum. L. subsp. durum). Protoplasma.

[38] Sun S (2008). TINY, a dehydration-responsive element (DRE)-binding protein-like transcription factor connecting the DRE- and ethylene-responsive element-mediated signaling pathways in Arabidopsis. J Biol Chem.

[39] Park JM (2001). Overexpression of the tobacco Tsi1 gene encoding an EREBP/AP2-type transcription factor enhances resistance against pathogen attack and osmotic stress in tobacco. Plant Cell.

[40] Wang H (2004). Ectopic overexpression of tomato JERF3 in tobacco activates downstream gene expression and enhances salt tolerance. Plant Mol Biol.

[41] Wang Y (2012). An ethylene response factor OsWR1 responsive to drought stress transcriptionally activates wax synthesis related genes and increases wax production in rice. Plant Mol Biol.

[42] Vanholme R, Morreel K, Ralph J, Boerjan W (2008). Lignin engineering. Curr Opin Plant Biol.

[43] Li L (2003). Combinatorial modification of multiple lignin traits in trees through multigene cotransformation. Proc Natl Acad Sci USA.

[44] Li YH (2003). BRITTLE CULM1, which encodes a COBRA-like protein, affects the mechanical properties of rice plants. Plant Cell.

[45] Sindhu A (2007). Maize Brittle stalk2 encodes a COBRA-like protein expressed in early organ development but required for tissue flexibility at maturity. Plant Physiology.

[46] Hu WJ (1999). Repression of lignin biosynthesis promotes cellulose accumulation and growth in transgenic trees. Nat Biotechnol.

[47] Novaes E, Kirst M, Chiang V, Winter-Sederoff H, Sederoff R (2010). Lignin and biomass: a negative correlation for wood formation and lignin content in trees. Plant Physiol.

[48] Dhugga KS (2007). Maize biomass yield and composition for biofuels. Crop Sci.

[49] Zhu X (2010). Virus-induced gene silencing offers a functional genomics platform for studying plant cell wall formation. Mol Plant.

[50] Nieminen KM, Kauppinen L, Helariutta Y (2004). A weed for wood? Arabidopsis as a genetic model for xylem development. Plant Physiol.

[51] Studer RA, Robinson-Rechavi M (2009). How confident can we be that orthologs are similar, but paralogs differ?. Trends Genet.

[52] Lavoie H (2010). Evolutionary tinkering with conserved components of a transcriptional regulatory network. PLoS Biol.

[53] Kannangara R (2007). The transcription factor WIN1/SHN1 regulates Cutin biosynthesis in Arabidopsis thaliana. Plant Cell.

[54] Kolosova N (2004). Isolation of high-quality RNA from gymnosperm and angiosperm trees. Biotechniques.

[55] McCarthy RL, Zhong R, Ye ZH (2011). Secondary wall NAC binding element (SNBE), a key cis-acting element required for target gene activation by secondary wall NAC master switches. Plant Signal Behav.

[56] Ohme-Takagi M, Shinshi H (1995). Ethylene-inducible DNA binding proteins that interact with an ethylene-responsive element. Plant Cell.

[57] Livak KJ, Schmittgen TD (2001). Analysis of relative gene expression data using real-time quantitative PCR and the 2(-Delta Delta C(T)) Method. Methods.

[58] Whiting P, Favis BD, St-germain FGT, Goring DAI (1981). Fractional Separation of Middle Lamella and Secondary Wall Tissue From Spruce Wood. Journal of Wood Chemistry and Technology.

[59] Ji XY (2014). A Transient Transformation System for the Functional Characterization of Genes Involved in Stress Response. Plant Mol Biol Rep.

[60] Yoshizumi T, Nagata N, Shimada H, Matsui M (1999). An Arabidopsis cell cycle -dependent kinase-related gene, CDC2b, plays a role in regulating seedling growth in darkness. Plant Cell.

[61] Desikan R, Hagenbeek D, Neill SJ, Rock CD (1999). Flow cytometry and surface plasmon resonance analyses demonstrate that the monoclonal antibody JIM19 interacts with a rice cell surface component involved in abscisic acid signalling in protoplasts. FEBS Lett.

