# Supplementary material for: Functional Characterization of Populus PsnSHN2 in Coordinated Regulation of Secondary Wall Components in Tobacco
Source: Sci Rep. 2017 Mar 3;7:42. doi: 10.1038/s41598-017-00093-z (PMC5428377; doi:10.1038/s41598-017-00093-z)
Supplement: Supplementary file 1 — Supplementary file [file 41598_2017_93_MOESM1_ESM.pdf]

Functional Characterization of *Populus PsnSHN2* in Coordinated Regulation of Secondary Wall Components in Tobacco

Yingying Liu[1+], Minjing Wei[1+], Cong Hou[1], Tingting Lu[1], Lulu Liu, Hairong Wei[2], Yuxiang Cheng[1*] & Zhigang Wei[1*]

[1]State Key Laboratory of Tree Genetics and Breeding, Northeast Forestry University, Heilongjiang Harbin 150040, P. R. China
[2]School of Forest Resource and Environmental Science, Michigan Technological University, Houghton, MI 49931, USA

***Corresponding authors:** Yuxiang Cheng (chengyuxiang@nefu.edu.cn) & Zhigang Wei (zhigangwei1973@163.com)

+ These authors contributed equally to this work

93 *SHINE* genes from 44 species in the Figure 1B used for polygenetic analysis are listed below:

AcOSHN2-33054799 *(Ananas comosus*); *AhSHN2.1*-28845533, *AcOSHN2.2*-33054799 (*Arabidopsis halleri*); *AcOSHN2*-33054799 (*Ananas comosus*); *AhSHN2.1*-28845533, *AhSHN2.2*-28852433 (*Arabidopsis halleri*); *AlSHN2.1*-893032, *AlSHN2.2*-471733 (*Arabidopsis lyrata*); *AmhSHN2.1*-32833075, *AmhSHN2.2*-32829607 (*Amaranthus hypochondriacus*); *AmtSHN2.1*-31566663, *AmtSHN2.2*-31570236(*Amborella trichopoda*); *AtSHN1*-19655864, *AtSHN2*-19673076, *AtSHN3*-19671959 (*Arabidopsis thaliana*);*BdSHN2.1*-32803005, *BdSHN2.2*-32817090 (*Brachypodium distachyon*); *BrSHN2.1*-30614139, *BrSHN2.2*-30645401 (*Brassica rapa*); *BsSHN2.1*-30654831, *BsSHN2.2*-30679677 (*Boechera stricta*); *CcSHN2.1*-20793351, *CcSHN2.2*-20799880 (*Citrus clementina*); *CgSHN2.1*-28912583, *CgSHN2.*2-28918266 (*Capsella grandiflora*); *CpSHN2.1*-16415016 (*Carica papaya*); *CrSHN2.1*-20910567, *CrSHN2.2*-20907728 (*Capsella rubella*); *CsSHN2.1*-18113316, *CsSHN2.2*-18096347 (*Citrus sinensis*); *CuSHN2.1*-16976521, *CuSHN2.2*-16962497 (*Cucumis sativus*); *EgSHN2.1*-32039617, *EgSHN2.2*-32040256, *EgSHN2.3*-32037121, *EgSHN2.4*-32053465 (*Eucalyptus globulus*); *FvSHN2.1*-27266330, *FvSHN2.2*-27244461 (*Fragaria vesca*); *GmSHN2*-30554023 (*Glycine max*); *GrSHN2.1*-26757988, *GrSHN2.2*-26776464 (*Gossypium raimondii*); *KlSHN2.1*-35740793, *KlSHN2.2*-35740315 (*Kalanchoe laxiflora*); *KmSHN2.1*-32590261, *KmSHN2.2*-32588799 (*Kalanchoe marnieriana*); *LuSHN2.1*-23172553, *LuSHN2.2*-23175361 (*Linum usitatissimum*); *MdSHN2.1*-22657051, *MdSHN2.2*-22673084 (*Malus domestica*); *MeSHN2.1*-32365075, *MeSHN2.2*-22673084 (*Manihot esculenta*); *MgSHN2.1*-28930189, *MgSHN2.2*-28940082 (*Mimulus guttatus*); *MtSHN2.1*-31059192 (*Medicago truncatula*); *OsSHN2.1*-33135229, *OsSHN2.*1-33146673 (*Oryza sativa*); *OtSHN2.1*-36000775, *OtSHN2.2*-36015486 (*Oropetium thomaeum*); *PhpSHN2.1*-32899668, *PhpSHN2.2*-32960909 (*Physcomitrella patens*); *PhSHN2.1*-32525402, *PhSHN2.2*-32483837 (*Panicum hallii*); *PpSHN2.1*-32119127, *PpSHN2.2*-32104465 (*Prunus persica*); *PsnSHN2* (*Populus simonii* ×*Populus nigra*); *PtrSHN2.1*-27009360, *PtrSHN2.2*-27005079, *PtrSHN2.3*-27010911, *PtrSHN2.4*-27006074, *PtrSHN2.5*-27000016, *PtrSHN2.6*-26996731 (*Populus trichocarpa*); *PvSHN2.1*-35854375, *PvSHN2.2*-30249925 (*Panicum virgatum*); *SBSHN2.1*-32746920, *SBSHN2.2*-32725971 (*Sorghum bicolor*); *SfSHN2.1*-32614786, *SfSHN2.2*-32620753 (*Sphagnum fallax*); *SlSHN2.1*-27291745, *SlSHN2.2*-27306704 (S*olanum lycopersicum*); *SmSHN2*-15417654 (*Selaginella moellendorffii*); *SpSHN2.1*-31426381, *SpSHN2.2*-31426375 (*Salix purpurea*); *StSHN2*-24404241 (*Solanum tuberosum*); *SvSHN2.1*-32665202, *SvSHN2.2*-32648351 (*Setaria viridis*); *TaSHN2.1*-31963741, *TaSHN2.1*-31968178 (*Triticum aestivum*); *TcSHN2.1*-27426888, *TcSHN2.2*-27430158 (*Theobroma cacao*); *TpSHN2.1*-35979517, *TpSHN2.2*-35964739 (*Trifolium pratense*); *VvSHN2.1*-17826288, *VvSHN2.2*-17840938 (*Vitis vinifera*); *ZaSHN2.1*-33179439, *ZaSHN2.2*-33176470 (*Zostera marina*); *ZmSHN2.1*-30984872, *ZmSHN2.2*-31024932 (*Zea mays*).

Table S1 The primers used to amplify *SHN2* from *Populus simonii* ×*Populus nigra* (*PsnSHN2*)

Gene Name	Primers	Sequence(5'-3')
PsnSHN2	PsnSHN2cDNAF	ATGGTACAATCAAAGAAATTCAGAG
	PsnSHN2cDNAR	TCAATTCCGATTAAGCAGCTC

Table S2 The primers used to amplify *PsnSHN2* full-length coding region without the termination codon

Gene Name	Primers	Sequence(5'-3')
PsnSHN2	PsnSHN2subF	CACCATGGTACAATCAAAGAAATTCAGAG
	PsnSHN2subR	ATTCCGATTAAGCAGCTC

Table S3 The primers used to amplify *PsnSHN2* sequence for transcriptional activation analysis

Gene Name	Primers	Sequence(5'-3')
PsnSHN2	PsnSHN2BDF	CATGGAGGCCGAATTCATGGTACAATCAAAGAAATTCAGAG
	PsnSHN2BDR	GCAGGTCGACGGATCCTCAATTCCGATTAAGCAGCTC

Note: The dash line is fusion site while the underline represents an enzyme digestion site.

Table S4 The primers and *cis*-acting element sequences used in the yeast one-hybrid analysis

		Sequence(5'-3')
PsnSHN2	PsnSHN2ADF	TGGCCATTATGGCCCGGGATGGTACAATCAAAGAAATTCAGAG
	PsnSHN2ADR	GACATGTTTTTTCCCGGGTCAATTCCGATTAAGCAGCTC
SNBE-F		AATTCTCCTTTTCTCTCTAAGCATTCCTTTTCTCTCTAAGCATTCCTTTTCTCTCTAAGCATGAGCT
SNBE-R		CATGCTTAGAGAGAAAAGGAATGCTTAGAGAGAAAAGGAATGCTTAGAGAGAAAAGGAG
SMRE-F		AATTCACCAAATACCAAATACCAAATACCAAATACCAAATACCAAATACCAAATACCAAATACCAAATGAGCT
SMRE-R		CATTTGGTATTTGGTATTTGGTATTTGGTATTTGGTATTTGGTATTTGGTATTTGGTATTTGGTG
AC-Ⅰ-F		AATTCACCTACCACCTACCACCTACCGAGCT
AC-Ⅰ-R		CGGTAGGTGGTAGGTGGTAGGTG
AC-Ⅱ-F		AATTCACCAACCACCAACCACCAACCGAGCT
AC-Ⅱ-R		CGGTTGGTGGTTGGTGGTTGGTG
GCC box -F		AATTCGCCGCCGCCGCCGCCGCCGAGCT
GCC box -R		CGGCGGCGGCGGCGGCGG

Note: The dash line is fusion site while the underline represents an enzyme digestion site.

Table S5 The primers used in the *PsnSHN2* overexpression transgenic tobacco (*Nicotiana tabacum*)

Gene Name	Primers	Sequence(5'-3')
PsnSHN2	PsnSHN2overexF	CGGGATCCATGGTACAATCAAAGAAATTCAGAG

	PsnSHN2overexR	GGGGTACCTCAATTCCGATTAAGCAGCTC
	pROKIIF	AGACGTTCCAACCACGTCTT
	pROKIIR	CCAGTGAATTCCCGATCTAG

Note: The underline represents an enzyme digestion site.

Table S6 The primers used in quantitative RT-PCR analysis of *P. simonii* ×*P. nigra* and *PsnSHN2* lines

Gene Name	Primers	Sequence(5'-3')
PsnSHN2	PsnSHN2RTF	TTCGCCACCCATTACTGAAGAG
	PsnSHN2RTR	GACTTGTCGGGAAATTGGTCTT
PsnActin1	Actin1F	AGGCAGGTTTCGCAGGAGATGA
	Actin1R	TCCATCACCAGAATCCAGCACA
NtActin2	Actin2F	ATCGCCATTGTTCTTTC
	Actin2R	TGTCTCCGTTCTTCACTTC

Table S7 The primers used to analyze expression levels of the transcription factors and biosynthesis genes involved in secondary wall formation of *PsnSHN2* lines

Gene Name	Accession number	Primers	Sequence(5'-3')
NtCesA4	JN009108.1	NtCesA4RTF	TATGGATACGGGACTGTTGCATGG
		NtCesA4RTR	TTACCACCACCTTTACTGCCTCCG
NtCesA7	JQ735443.1	NtCesA7RTF	TTGCTTCGTCCAGTTTCCTCAAAG
		NtCesA7RTR	CTGGACCTTGTATTCCGTCTAGGC
NtCesA8	JQ735445.1	NtCesA8RTF	CCAAAGCTGCAGATGATGGAGAGT
		NtCesA8RTR	TTGAGTGCATCAGAGAAACCAGCA
NtIRX8	JQ735447.1	NtIRX8RTF	TGAGCATTCTACCAATTCCAATGC
		NtIRX8RTR	CTGCACGAGTGATGAAGCAACAAC
NtIRX9	JQ735449.1	NtIRX9RTF	ACATGGCCAATGGCATTATTATCT
		NtIRX9RTR	CACTTGAAACTCTAATTGGAGGCC
NtIRX10	JQ735450.1	NtIRX10RTF	CCCTGTTTACACCACTTGTGACCT
		NtIRX10RTR	AGAAATGATCAGCTCCCTCTGTCC
NtPAL1	M84466.1	NtPAL1RTF	ATTGGAGCTTTCGAAGATGAATTG
		NtPAL1RTR	CTGTTCCAAGCTCCTTTCTCACAA
NtPAL4	X78269.1	NtPAL4RTF	CTGCAATTGCCAACAGGATAAAGG
		NtPAL4RTR	TGTCCATTGCACATTGCTGTGAAC
NtCAD14	X62343.1	NtCAD14RTF	CTGTTGGCCATCCTCTTGAACCTT
		NtCAD14RTR	CTTCCTGTGATGCTCTTTCTCCCG
NtCAD19	X62344.1	NtCAD19RTF	TGATACTGTCCCTGTTGGCCATCC
		NtCAD19RTR	TTCCAGTGATGCTCTTTCTCCCGA
Nt4CL1	U50845.1	Nt4CL1RTF	ATGGTTACACACTGGCGACATTGG
		Nt4CL1RTR	ACAGCAGCATCAGAAATGTTGGGA
Nt4CL2	U50846.1	Nt4CL2RTF	TGAGCTCGAAGCTCTCCTTCTCAA
		Nt4CL2RTR	CTTCAGTAATGGTGGATCCGTTGG
NtHCT	AJ507825.1	NtHCTRTF	ACTCCTATTGCAGTCGCAGGTGAT
		NtHCTRTR	ACGTATGTGCACCACGAACAAGAG
NtCCoAOMT	U38612.1	NtCCoAOMTRTF	CCTGAGCCCATGAAAGAGCTAAGA

		NtCCoAOMTRTR	CAATCTCCATTGTGTTCTTGGCAT
Nt4CL3	XM_009605419.1	Nt4CL3RTF	TAGTGTTGAAGCCCAGAAAGC
		Nt4CL3RTR	TGTCCACTGCTGCCTGCTAT
NtVND6	XM_009592752.1	NtVND6RTF	GTGTTCCTCCAGGTTTTAGATTTC
		NtVND6RTF	CTATTTGTCCTCGTTCCAGTCG
NtSND1	XM_009776136.1	NtSND1RTF	ATGGGACATTCAAGAGAAGTGC
		NtSND1RTR	TGTGTATGACTTTATCACGACCTG
NtMYB83	XM_009613740.1	NtMYB83RTF	CCTCAGGCAAGAACGGGA
		NtMYB83RTR	CTTAGCCTACAACTCTTCCCACA
NtMYB20	XM_009622695.1	NtMYB20RTF	GTAAAGAAAGGTCCATGGTCAGC
		NtMYB20RTR	CCCCTCTTCAAATCTGGTCTTAG
NtMYB43	XM_009591401.1	NtMYB43RTF	TTGTTGAAATAATAATGGGGAGAC
		NtMYB43RTR	CAGTCTACAACTCTTTCCACACCT
NtMYB58	XM_009594824.1	NtMYB58RTF	GGTGTGGAAAGAGTTGCCG
		NtMYB58RTR	CTTTGACCACCTGTTCCCC
NtMYB85	XM_009596755.1	NtMYB85RTF	TCTTCGATGGACTAATTACCTTCAC
		NtMYB85RTR	TTATCAGTCCTTCCTGGTAATCTTA
NtMYB63	XM_009613943.1	NtMYB63RTF	TATTGAGGGAGATGAGAAAAGGG
		NtMYB63RTR	TTTCCACGCCTCAGTAGCC

Table S8 The primers used to amplify proximal promoter regions of transcription factors from *P. simonii ×P. nigra* that were used in transactivation analysis

Gene Name	Primers	Sequence(5'-3')
PsnWND1A	PsnWND1AproF	ATGCCTTCAGATCATAATTACTAGT
	PsnWND1AproR	TTTTGCTAGGACAAATTTATTGTAT
PsnWND3A	PsnWND3AproF	GCAGTTTTTTTTGTTCGCCTT
	PsnWND3AproR	TTCTGATCCTGCTAGGCCTT
PsnMYB28	PsnMYB28proF	GGCTAGTGCCTCGATGTCATG
	PsnMYB28proR	GATCTCTCTCTCTCTCTCTCTCTCTCC
PsnMYB85	PsnMYB85proF	GGGTTAAAAAAAAATAATTGCGATC
	PsnMYB85proR	ATTGCACAGAGTAGCTAGCTAGCTG
PsnMYB192	PsnMYB192proF	AGGGTAGAGTCATAAAGAGAGAATA
	PsnMYB192proR	GATATGATCTCTCTCAAATTTGCTA
PsnMYB3	PsnMYB3proF	ACTGTCCTGGATGACCAACTC
	PsnMYB3proR	TGTGTTTGATACTCTGATGATAGGC
PsnMYB20	PsnMYB20proF	GACCCTTCTCTTTCCTCTTGTT
	PsnMYB20proR	CTTTCACAAGATCACAAACACC
PsnMYB152	PsnMYB152proF	TTTTCCCAAGAAGGGCTGT
	PsnMYB152proR	CTTTCACGAACACCAAGATGTA

Table S9 The frequency of occurrence of *cis*-acting elements present in the proximal promoter regions of poplar (*Populus trichocarpa*) transcription factors that are involved in the hierarchical transcriptional network governing wood formation

***Cis*-acting**	**Sequence**	**Gene Name(ortholog**	**Accession**	**Frequency of**

element name		of *Arabidopsis*)	number	occurrence
SNBE	(T/A)NN(C/T)(T/C/G)TNNNNNNNA(A/C)GN(A/C/T)(A/T)	*PtrWND1A(SND1)*	Potri.011G153300	6
		PtrWND3A(VND6)	Potri.015G127400	2
		PtrMYB28(MYB58)	Potri.007G067600	2
		PtrMYB85(MYB85)	Potri.003G114100	4
		PtrMYB192(MYB63)	Potri.005G096600	4
		PtrMYB3(MYB83)	Potri.001G267300	6
		PtrMYB20(MYB20)	Potri.004G086300	5
		PtrMYB152(MYB43)	Potri.017G130300	2
SMRE	ACC(A/T)A(A/C)(T/C)	*PtrMYB152(MYB43)*	Potri.017G130300	2
		PtrMYB3(MYB83)	Potri.001G267300	1
AC-Ⅰ	ACCTACC	*PtrMYB3(MYB83)*	Potri.001G267300	2
		PtrMYB85(MYB85)	Potri.003G114100	1
AC-Ⅱ	ACCAACC	*PtrMYB3(MYB83)*	Potri.001G267300	1
GCC box	GCCGCC	*PtrWND3A(VND6)*	Potri.015G127400	1
		PtrMYB152(MYB43)	Potri.017G130300	1

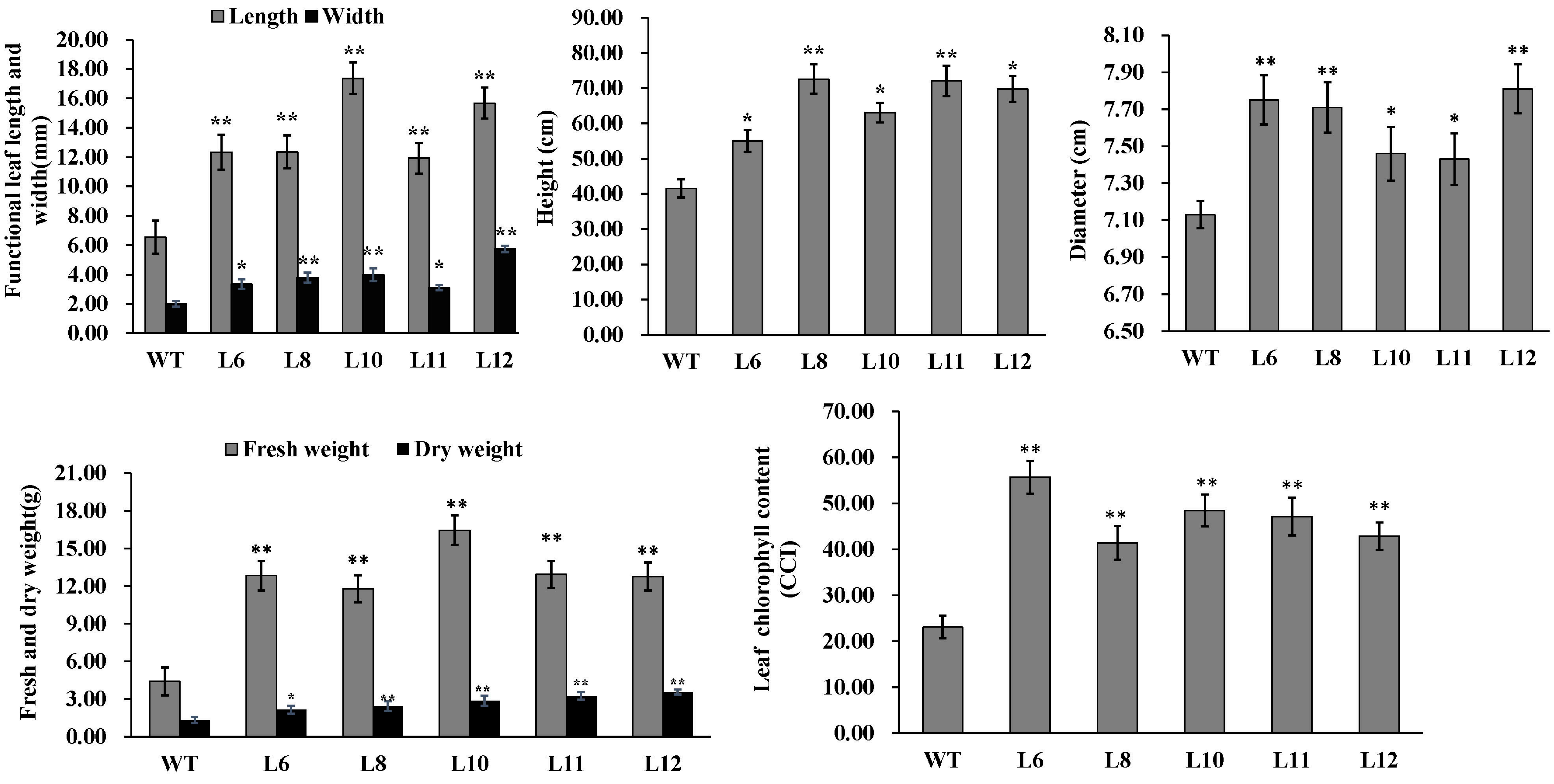

Length
Width
Functional leaf length and width(mm)
20.00
18.00
16.00
14.00
12.00
10.00
8.00
6.00
4.00
2.00
0.00
WT
L6
L8
L10
L11
L12
Height (cm)
90.00
80.00
70.00
60.00
50.00
40.00
30.00
20.00
10.00
0.00
Diameter (cm)
8.10
7.90
7.70
7.50
7.30
7.10
6.90
6.70
6.50
Fresh weight
Dry weight
Fresh and dry weight(g)
21.00
18.00
15.00
12.00
9.00
6.00
3.00
0.00
Leaf chlorophyll content (CCI)
70.00
60.00
50.00
40.00
30.00
20.00
10.00
0.00

Figure S1 Phenotypic changes in *PsnSHN2* transgenic lines. (A) Length and width of functional leaves of *PsnSHN2* transgenic lines and wild-type. (B) Heights of *PsnSHN2* lines and wild-type. (C) Stem diameters of *PsnSHN2* transgenic lines and wild-type at 3cm height above the root collar. (D) Fresh and dry weight of *PsnSHN2* transgenic lines and wild-type above ground. (E) Chlorophyll contents of *PsnSHN2* transgenic lines and wild-type. Error bars represent SD of three biological replicates. Asterisks indicate levels of significance of differential expression （ *t* test ; $*P<0.05$, $**P<0.01$).
